# DNA methylation differences in genes associated with human personal disorders and deviant behavior

**DOI:** 10.3934/Neuroscience.2024003

**Published:** 2024-03-25

**Authors:** I. B. Mosse, N. G. Sedlyar, K. A. Mosse, A. V. Kilchevsky

**Affiliations:** Laboratory of Human Genetics, the Institute of Genetics and Cytology, National Academy of Sciences of Belarus, Minsk, Republic of Belarus

**Keywords:** personal disorders, deviant behavior, epigenetic regulation, methylation of CpG sites, high-throughput sequencing

## Abstract

Epigenetic regulation of gene expression is involved in the progression of mental disorders, including deviant behavior, brain developmental, and personality disorders. The large number of genes has been studied for their activity association with stress and depression; however, the obtained results for the majority of these genes are contradictory. The aim of our study was to investigate the possible contribution of methylation level changes to the development of personality disorders and deviant behavior. A systematic study of CpG Islands in 21 target regions, including the promoter and intron regions of the 12 genes was performed in DNA samples extracted from peripheral blood cells, to obtain an overview of their methylation status. High-throughput sequencing of converted DNA samples was performed and calling of the methylation sites on the “original top strand” in CpG islands was carried out in the Bismark pipeline. The initial methylation profile of 77 patients and 48 controls samples revealed a significant difference in 7 CpG sites in 6 genes. The most significant hypermethylation was found for the target sites of the *HTR2A* (p-value = 1.2 × 10^−13^) and *OXTR* (p-value = 2.3 × 10^−7^) genes. These data support the previous reports that alterations in DNA methylation may play an important role in the dysregulation of gene expression associated with personality disorders and deviant behavior, and confirm their potential use as biomarkers to improve thediagnosis, prognosis, and assessment of response to treatment.

## 1. Introduction

Psychiatric disorders, including stress and depression, arise due the combined action of many factors including neurobiology, genetics, living conditions, and childhood experiences. Recent researches confirmed that epigenetic mechanisms play an important role in the development of psychiatric conditions [1]–[3].

Epigenetic regulation of gene expression by DNA methylation, the modification of histones, and small RNAs are the most likely mechanism of action of environmental factors, such as stress, having a lifelong impact on neuropsychiatric state. In fact, stress with the involvement of epigenetic mechanisms is being considered as one of the basic factors that induce psychiatric disorders [4]–[6]. Nowadays, psychiatric and behavioral epigenetics is a separate and promising area of research.

DNA methylation is a well-studied epigenetic modification which represents the addition of a CH3 group to a cytosine. It is a highly stable epigenetic mark that usually takes place at CpG sites and can be preserved throughout the human lifetime. Such epigenetic regulation generally exerts a negative effect on level of transcription; however, in some cases, DNA methylation may enhance gene expression. The most prevalent mechanism of gene expression inhibition is through the locking of DNA sequences to prevent the identification and binding of activating transcription factors.

A great number of genes have been investigated for their association with stress and depression; however, the research data for most of these genes were contradictory. Recently published reviews [7]–[11] have reported the associations between depression and DNA methylation changes in several genes, including *BDNF, SLC6A4, NR3C1, FKBP5, OXTR*, and *MAOA*.

Previous studies noted that genes associated with depression mainly related to the hypothalamic-pituitary-adrenal (HPA) axis [5] and the 5-hydroxytryptamine system (5-HT) [6]. Depressive patients have significantly increased levels of corticotropin releasing hormone due to the decreased function of the glucocorticoid receptor (GR) encoded by *NR3C1*. In addition, gene expression of *FKBP5* could be activated by GR signaling.

The main 5-hydroxytryptamine system genes with possible methylation alterations include the brain derived neurotrophic factor gene (*BDNF*), the serotonin transporter gene (*SLC6A4*), hydroxytryptamine receptors (*HTR*), and monoamine oxidase A (*MAOA*) [6]. Although previous studies have stated that DNA methylation of 5-HT and the HPA axis are associated with either depressive symptoms or depressive disorders, consistent evidence for the association is limited.

In this study, we investigated if DNA methylation of genes involved in the progression of psychiatric disorders is altered in individuals with personality disorders and deviant behavior. A systematic study of CpG islands (CGIs) in the promoter and intron regions of the genes was performed to get an overview of their methylation status.

## 2. Materials and methods

The study was carried out on 2 groups of representatives of the Belarussian population. The patients group from the Republican Scientific and Practical Center for Mental Health suffering from personality disorders and deviant behavior consisted of 67 (87%) men and 10 (13%) women, with a mean age of 41 years (range 22–69) at first blood sampling. The control group of relatively healthy volunteers contained 48 people matched for gender and age.

Informed consent for inclusion in the study was obtained from each participant in accordance with the Declaration of Helsinki (as amended in 2013). The consent form and the study program were approved by the Bioethics Committee of the Institute of Genetics and Cytology at the National Academy of Sciences of Belarus.

Patients included in our study were treated at the Republican Scientific and Practical Center for Mental Health with the following diagnosis according to the International Classification of Diseases (ICD) 10: persons with affective and neurotic disorders (F31–F48). The main impairment of these disorders is a change in emotions and mood towards either depression (with or without anxiety) or elation. Changes in mood are usually accompanied by changes in the overall activity level. Most other symptoms are either secondary or easily explained by changes in mood and activity. Such disorders most often tend to recur, and the onset of an individual episode can often be associated with stressful events and situations.

Genomic DNA was extracted from the whole blood using a sorbent-based extraction kit; DNA samples were subjected to the bisulfite conversion of each unmethylated cytosine to uracil via deamination. Bisulfite conversion of 1 µg of DNA was performed using the EpiTect Fast DNA Bisulfite Kit (Qiagen). After conversion, 300–500 base pairs fragments of genomic DNA were obtained, with some quantities of more than 500 base pairs. The quantity and quality of the converted DNA samples were determined with the Agilent 4200 TapeStation (Agilent).

Primer pairs were specifically designed for the CGIs in the promoter and intron regions of the gene under study. A total of 21 target regions of the 12 studied genes were amplified from converted DNA samples.

The quality and concentration of the PCR products were determined on a GloMax Explorer (Promega) fluorometer with a QuantiFluor® ONE dsDNA System (Promega) reagent kit.

Next, all amplicons of each subject were normalized and combined into an equimolar pool using an Eppendorf epMotion liquid handling system. The NGS libraries were prepared using the QIAseq Methyl Library Kit (Qiagen). The size selection of fragments was performed using AMPure XP beads (Beckman Coulter) and the concentration of the subject libraries was measured with the Agilent 4200 TapeStation (Agilent).

The library for loading was prepared according to the Illumina MiniSeq System Denature and Dilute Libraries Guide recommendations. The final concentration was empirically chosen as 8 pmol with a 10% PhiX control library.

High-throughput sequencing was performed on an Illumina MiSeq instrument with the v3 2x250 (500 cycles) Illumina - MiSeq Reagent Kit v3.

The quality of the reads was checked using the Fast QC program. Then, the low-quality bases were removed by trimming in Trimmomatic. Local mapping of sequencing fragments to the reference genome (GRCh37 [GCF_000001405.13]) was performed with the Burrows-Wheeler Aligner algorithm using the Bowtie2 software module in the Bismark utility.

Filtering followed by the transformation of the read alignment map (SAM), as well as sorting and indexing, was carried out in Samtools. Calling of the methylated sites on the “original top strand” in CpG islands was carried out in the Bismark pipeline. The ratio of demethylated to methylated sites was calculated using the Python programming language using the Pandas library.

The methylation levels of the individual CpG sites in the studied groups were compared using the Mann-Whitney criteria. Differences were accepted as statistically significant at p < 0.05.

**Table 1.** The amplicon locations and sizes of 21 target regions in the studied genes.

№	Gene and amplicon N	Position (Reference Genome Build hg19, Strand	Target Amplicon Length (bp)
1	*TRKB*	Chr9:84668523–84668590	68 bp
2	*CRH*	Chr8:66178523–66178594	72 bp
3	*OXTR*-1	Chr3:8769061–8769160	100 bp
4	*OXTR*-2	Chr3:8767614–8767659	46 bp
5	*FKBP5*-1	Chr6:35590602–35590701	100 bp
6	*FKBP5*-2	Chr6:35590648–35590757	110 bp
7	*FKBP5*-3	Chr6:35663934–35664005	72 bp
8	*NR3C1*	Chr5:143404032–143404124	93 bp
9	*BDNF*-1	Chr11:27700216–27700305	90 bp
10	*BDNF*-2	Chr11:27742305–27742408	104 bp
11	*BDNF*-3	Chr11:27720777–27720842	66 bp
12	*MAOA*	ChrX:43655941–43656026	86 bp
13	*SLC6A4*	Chr17:30236081–30236216	136 bp
14	*SKA2*	Chr17:59110331–59110398	68 bp
15	*CACNA1C*-1	Chr22:2230286–2230405	120 bp
16	*CACNA1C*-2	Chr12:2218746–2218860	115 bp
17	*COMT*-1	Chr12:19963698–19963788	91 bp
18	*COMT*-2	Chr12:19962318–19962410	93 bp
19	*HTR2A*-1	Chr13:46897303–46897383	81 bp
20	*HTR2A*-2	Chr13:46897282–46897375	94 bp
21	*HTR2A*-3	Chr13:46897493–46897642	150 bp

## 3. Results and discussion

The initial methylation profile of the 21 analyzed target regions in DNA samples from the patients and control group revealed a significant difference in DNA methylation in 7 CpG sites in 6 genes. The results are presented in Table 2.

**Table 2.** The degree of methylation of CpG sites with statistically in the groups of patients and controls. DNA methylation values are presented as average DNA methylation percentage per studied group.

CpG site	DNAm % patients	DNAm % control	Mann–Whitney Test, p-Value
*CACNA1C_15*__2339515	56.192	60.517	0.0190
*COMT_01*__19949901	75.430	73.016	0.0480
*FKBP5_38*__35558438	71.544	67.465	0.0042
*FKBP5_88*__35558488	96.949	96.186	0.0018
*HTR2A_06*__47471705	76.097	65.129	1.2×10^−13^
*NR3C1_47*__142783637	0.062	0.389	0.0240
*OXTR_07*__8810807	39.729	30.775	2.3×10^−7^

**Figure 1.**
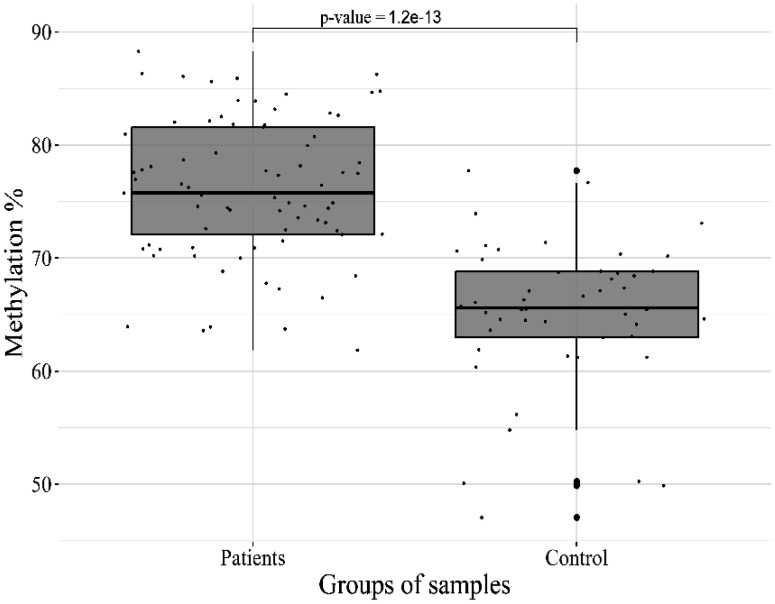
DNA methylation of *HTR2A*_06_47471705 CpG site in patients and controls.

**Figure 2.**
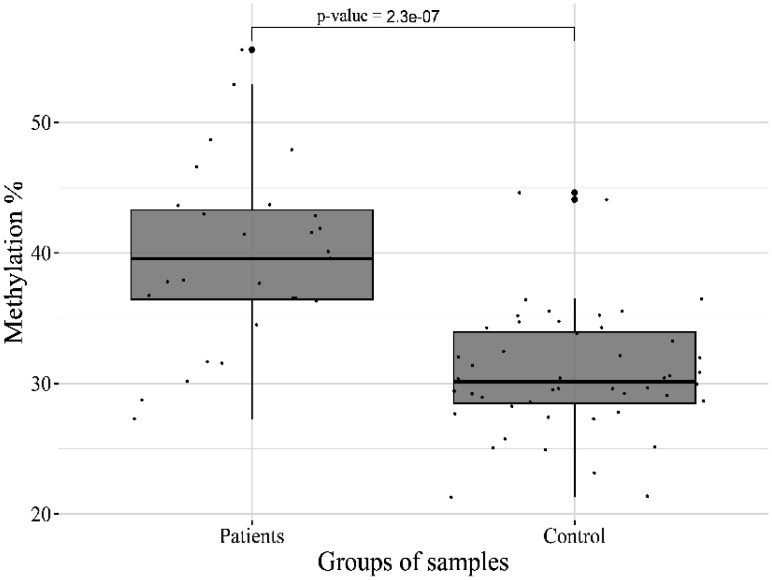
DNA methylation of *OXTR*_07__8810807 CpG site in patients and controls.

As can be seen from the table, the most significant hypermethylation was found for the target sites of the *HTR2A* (p-value = 1.2 × 10^−13^) and *OXTR* (p-value = 2.3 × 10^−7^) genes (Fig. 1,2)

The serotonin 2A receptor (*5-HT2A*) has been implicated in multiple psychiatric disorders. Serotonin acts as a neurotransmitter mainly in the gastrointestinal tract, blood, and different parts of the brain, thus influencing behavior and physiology. Many psychoactive drugs were found to act on serotonergic targets that made serotonin a perspective target in the study of mental disorders. Activation of *HTR2A* receptors in the gray region suppresses panic reactions, while their activation in the amygdala is more associated with generalized anxiety disorders and post-traumatic stress disorder [12].

Epigenetic modifications through methylation are now considered to be the primary mechanism of early life environmental influence on the behavioral phenotype development. Since embryogenesis, serotonin plays an important role in neurodevelopment, and the *5-HT2A* receptor promotes neuronal proliferation [13]. Thus, through environmental stressors, epigenetic changes in *HTR2A* expression may influence brain development and behavior. It has been established that methylation of placental *HTR2A* at CpG sites during the neonatal period is associated with a higher level of infant care [14].

Additionally, CpG hypermethylation in the promoter region has been found in adult patients with schizophrenia and bipolar disorder [15], thus suggesting that this may be the mechanism underlying the decrease in *HTR2A* expression in patients with schizophrenia.

DNA methylation of the oxytocin receptor gene (*OXTR*) is one of the epigenetic biomarkers of the oxytocin system. The oxytocin system is of significant interest in identifying biomarkers of mental disorders due to its involvement in many psychological conditions, the modulation of a wide range of physiological processes, and its potential use as a drug [16].

The *OXTR* gene structurally consists of four exons and three introns [17]. In its promoter, the gene contains a specific region with a large number of CpG sites, called MT2. It was discovered that the increased methylation of this region was associated with decreased *OXTR* expression, thus suggesting a regulatory role in gene transcription. [18]. *OXTR* is expressed in all regions of the human brain, with the highest levels of expression in the striatum.

In adults, variability in the methylation of CpG sites within the promoter has been shown to correlate with susceptibility to disorders associated with social deficits, as well as with individual differences in the brain's response to social information [19]. The greatest differences are observed for methylation sites -934, -924, and -901 (_8810807, _8810797, and_ 8810774, respectively) [20],[21].

Among other genes with observed significant differences in CpG sites methylation *NR3C1* was previously found to be associated with a significantly increased risk of depression in DNA methylation studies [22]. Similar to our study, patients with major depressive disorders had significantly lower methylation levels than healthy controls at 2 promoter CpG sites (p-value = 0,003).

NR3C1 encodes the glucocorticoid receptor (GR). In some previous studies, hypermethylation of NR3C1 was reported to be related to the reduction in GR with the subsequent HPA axis hyperactivation, thus casing the development of mental disorders and depression. It was suggested [22] that the majority of these studies focused on the relationship between NR3C1 methylation and early life adversities, rather than its clinical status. This is also true for later reports [23],[24]. Thus, this may indicate that the higher levels of NR3C1 methylation found in the aforementioned studies reflect the consequences of past childhood adversity, rather than the current psychiatric vulnerability.

**Figure 3.**
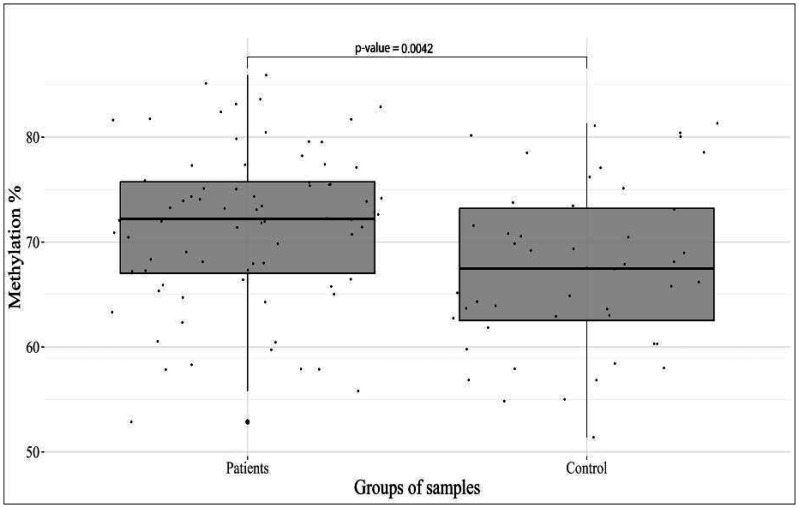
DNA methylation of *FKBP5*_38__35558438 CpG site in patients and controls.

Highly significant differences (p = 0.0042) between the studied groups were found in the methylation level of the *FKBP5_38*__35558438 CpG site (Fig. 3).

These results confirm the data of some publications that provide evidence of a connection between the expression of the *FKBP5* gene and various behavioral reactions, mental disorders, and depressive and aggressive conditions [25]–[27]. *FKBP5* modulates not only glucocorticoid receptor activity in response to stressors, but also a multitude of other cellular processes in both the brain and peripheral regions. Notably, the *FKBP5* gene is regulated via complex interactions among environmental stressors, *FKBP5* genetic variants, and epigenetic modifications of glucocorticoid-responsive genomic sites [26].

## 4. Conclusions

We found that hyper- and hypomethylations on promoter region CpG sites of 6 genes were significantly associated with psychiatric disorders.

Our study supports previous reports that changes in the DNA methylation level may play a significant role in the dysregulation of gene expression associated with personality disorders and deviant behavior, as well as confirm their potential use as biomarkers to improve the diagnosis, prognosis, and assessment of response to treatment.
